# The neural decoding toolbox

**DOI:** 10.3389/fninf.2013.00008

**Published:** 2013-05-22

**Authors:** Ethan M. Meyers

**Affiliations:** Department of Brain and Cognitive Sciences, McGovern Institute, Massachusetts Institute of TechnologyCambridge, MA, USA

**Keywords:** neural decoding, readout, multivariate pattern analysis, Matlab, data analysis, machine learning

## Abstract

Population decoding is a powerful way to analyze neural data, however, currently only a small percentage of systems neuroscience researchers use this method. In order to increase the use of population decoding, we have created the Neural Decoding Toolbox (NDT) which is a Matlab package that makes it easy to apply population decoding analyses to neural activity. The design of the toolbox revolves around four abstract object classes which enables users to interchange particular modules in order to try different analyses while keeping the rest of the processing stream intact. The toolbox is capable of analyzing data from many different types of recording modalities, and we give examples of how it can be used to decode basic visual information from neural spiking activity and how it can be used to examine how invariant the activity of a neural population is to stimulus transformations. Overall this toolbox will make it much easier for neuroscientists to apply population decoding analyses to their data, which should help increase the pace of discovery in neuroscience.

## Introduction

Population decoding is a data analysis method in which a computer algorithm, called a “pattern classifier,” uses multivariate patterns of activity to make predictions about which experimental conditions were present on particular trials (Bialek et al., 1991; Oram et al., 1998; Dayan and Abbott, 2001; Sanger, 2003; Brown et al., 2004; Quian Quiroga and Panzeri, 2009; Meyers and Kreiman, 2012). For example, a classifier could use the pattern of firing rates across a population of neurons to make predictions about which stimulus was shown on each trial. By examining how accurately the classifier can predict which experimental conditions are present, one can assess how much information about the experimental variables is in a given brain region, which is useful for understanding the brain region's function (Quian Quiroga and Panzeri, 2009). Additionally, one can use population decoding to examine more complex questions about how neural activity codes information across time and whether information is contained in abstract/invariant format (Hung et al., 2005; Meyers et al., 2008, 2012; Crowe et al., 2010). Because it is difficult to address these more complex questions using the most common data analysis methods, increasing the use of population decoding methods should lead to deeper insights and should help speed up the pace of discovery in neuroscience.

Currently population decoding is widely used in brain-computer interfaces (Schwartz et al., 2001; Donoghue, 2002; Nicolelis, 2003) and to analyze fMRI data (Detre et al., 2006; Haynes and Rees, 2006; O'Toole et al., 2007; Mur et al., 2009; Pereira et al., 2009; Tong and Pratte, 2012), however, it is still infrequently used when analyzing electrophysiology data from most neural system. One likely reason that population decoding methods are not widely used is due to the fact that running a decoding analysis requires a fair amount of knowledge of machine learning and computer programming. In order to make it easier for neuroscientists to apply population decoding analyzes to their data, we have created the Neural Decoding Toolbox (NDT). The toolbox is implemented in Matlab, a language that is widely used by neuroscientists, and is designed around a set of abstract object classes that allow one to extend its functionality. The toolbox can be applied to data from many different types of recording modalities (e.g., neural spiking data, local field potentials, magneto/electro-encephalographic signals, etc.), and the only requirement is that an experiment has been run in which the same experimental trials were repeated a few times and that data is available from multiple recording sites^1^^,^^2^. Using the objects provided by the toolbox, one can easily compare neural representations across time and across stimulus transformations which allows one to gain deeper insights into how information is coded in neural activity. Below we describe the population decoding process in more detail, outline the structure of the toolbox, and we give some examples of how it can be used to explore questions related to neural representations.

## Basics of pattern classification

As described briefly above, a pattern classifier is an algorithm that takes multivariate data points, and attempts to predict what experimental condition was present when each data point was recorded (Vapnik, 1999; Poggio and Smale, 2003). In order for the classifier to be able to make these predictions, a two-step process is typically used (Duda et al., 2001). In the first “training step,” the classifier is given a subset of the data called the “training set,” which contains examples of patterns of activity from a number of trials, and a set of labels that list the experimental condition that was present on each of these trials. The classifier algorithm then “learns” a relationship between these patterns of neural activity and the experimental conditions such that the classifier can make predictions about which experimental condition is present given a new pattern of neural activity. In the second “test” step, the ability of the classifier to make correct predictions is assessed. This is done by having the classifier make predictions about experimental conditions using data that was not included in the training set, and then assessing how accurate these predictions are by comparing them to the actual experimental conditions that were present when the experiment was originally run. To gain robust estimates of the classification accuracy, a cross-validation procedure is often used in which a dataset is divided into *k* different splits, and the classifier is trained using data from *k* − 1 of these splits, and tested on data from the remaining split; this procedure is repeated *k* times using a different test split each time, which generates *k* different estimates of the classification accuracy, and the final classification accuracy is the average of these results.

## The design of the neural decoding toolbox

In order to implement the classification procedure in a flexible way, the NDT is designed around four abstract object classes that each have a particular role in the decoding procedure. The four object types are:

1. *Datasources*. These objects generate training and test splits of data. Datasource objects must have a method get_data that returns the training and test splits of data and labels.
2. *Feature-preprocessors*. These objects learn preprocessing parameters from the training data, and apply preprocessing to the training and test data (prior to the data being sent to the classifier). Feature-preprocessor objects must have a set_properties_with_training_data method that takes training data and labels, sets the preprocessing parameters based on the training data, and returns the preprocessed training data. These objects must also have a preprocess_test_data method that takes the test data and applies processing to it, and a method get_current_info_to_save which allows the user to save extra information about the preprocessing parameters^3^.
3. *Classifiers*. These objects build a classification function from the training data, and make predictions on the test data. Classifier objects must have a train method that takes the training data and labels and learns parameters from them, and a test method, that takes test data and makes predictions about which classes the data points belongs to.
4. *Cross-validators*. These objects run a cross-validation loop which involves retrieving data from the datasource, applying preprocessing to the data, training and testing a classifier, and calculating measures of decoding accuracy. Cross-validator objects must have a method run_cv_decoding which returns decoding measures from running a cross-validation procedure using specified preprocessors, a classifier and a datasource object.

**Figure 1:**
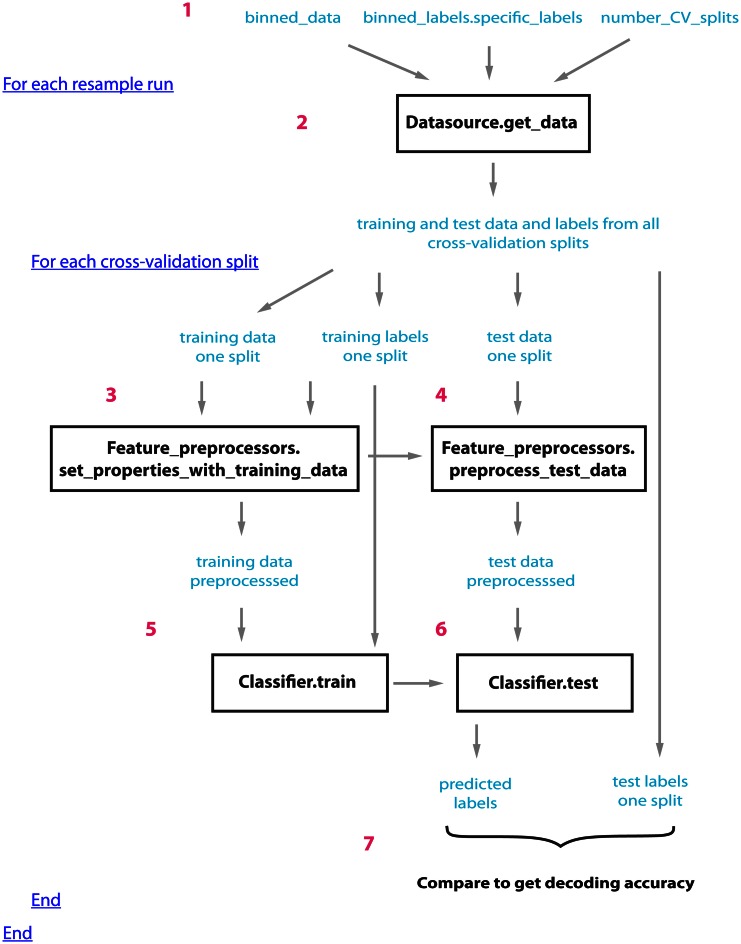
**An outline of how the datasource (DS), feature-preprocessors (FP), and classifier (CL) interact within the standard_resample_CV object's run_cv_decoding method.** Prior to calling the cross-validator's run_decoding method, a datasource object must be created that takes binned_data, specific binned_labels, and the number of cross-valdations splits as inputs (step 1); (a classifier and feature preprocessor objects must also be created and passed to the CV object). The standard_resample_CV’s run_cv_decoding method contains two major loops, the first loop calls the datasource's get_data method to generate all the training and test cross-validation data splits (step 2), while the second loop runs through each cross-validation split and assesses the decoding accuracy based on the data in each split (steps 3–6) as follows: First the training data and labels are passed to the feature preprocessor objects (step 3) which preprocesses the training data and learns the parameters necessary to preprocess the test data. The test data is then preprocessed using these learned parameters (step 4). The preprocessed training data along with the labels are passed to the classifier object which learns the relationship between the training data and the labels (step 5). The test data is then passed to the trained classifier, which makes predictions about what experimental conditions were present using this data (step 6). The predictions of the classifier are compared to the actual experimental conditions that were present to determine whether there is a reliable relationship between the data and particular experimental conditions (step 7). The whole process is repeated a number of times (outer loop at step 2) using different data splits in order to get a robust estimate of the decoding accuracy. In order to create the full temporal-cross-training matrix, two additional loops are run inside the inner loop that are involved in training and testing the classifier at all possible time periods (these loops are not shown in this figure). Figure A1 gives pseudo-code describing this process.

**Figure A1:**
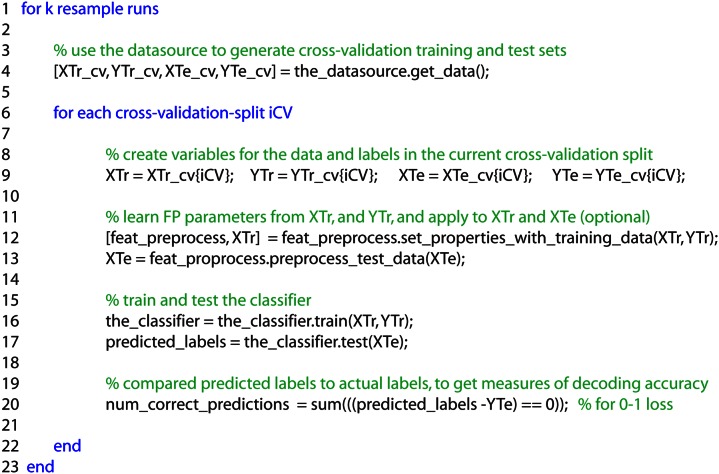
**An outline of how the datasource (DS), feature-preprocessors (FP), and classifier (CL) interact within the cross-validator (CV) object's run_cv_decoding method.** The the_cross_validator.run_cv_decoding method works by first generating training and test cross-validation splits using the datasource the_datasource.get_data method (line 4). For each cross-validation split, feature-preprocessing is applied to the data (line 12), and a classifier is trained and tested on this preprocessed data (lines 16–17). The accuracy of the classifier's predictions are assessed (line 20), and this whole procedure can be repeated multiple times (line 1), generating new training and test splits (and potentially also pseudo-populations) on each run. Note: XTr_cv and YTr_cv refer to the *training* data and labels from all cross-validation splits, and XTe_cv and YTe_cv refer to the *test* data and labels from all cross-validation splits. Similarly, XTr, YTr, XTe, and YTe, refer to the training and test data and labels from one particular cross-validation split.

Figure 1 illustrates how the datasource, feature-preprocessors, and classifier interact within cross-validator's run_cv_decoding method, and Figure A1 gives pseudo-code outlining their interactions.

**Figure 2:**
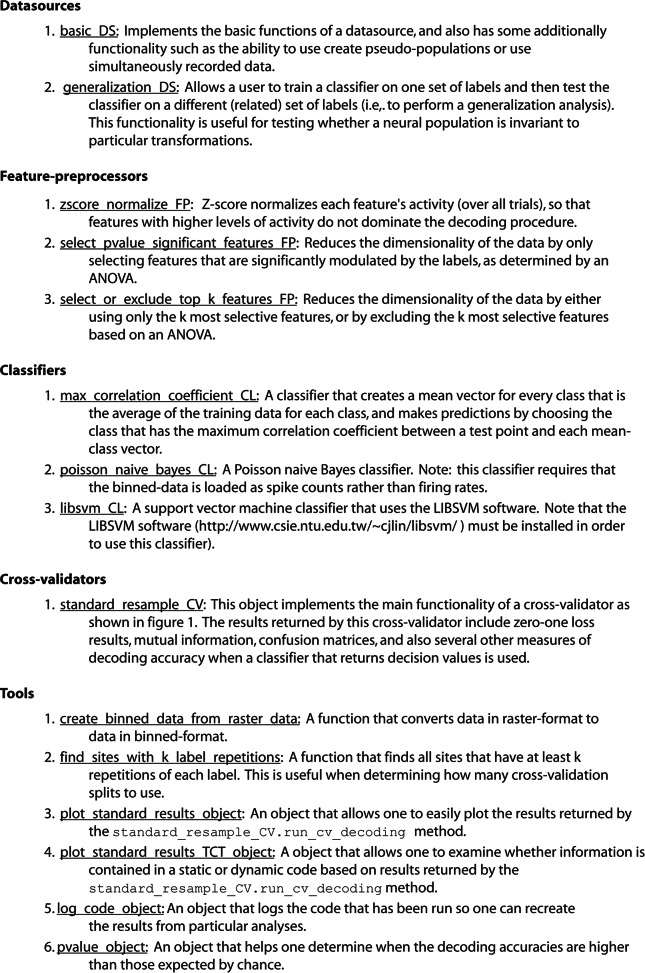
**A list of the datasource, classifier, feature-preprocessor, cross-validator objects and helper tools that come with version 1.0 of the NDT**.

By defining clear interfaces for these four abstract object classes one can flexibly exchange particular parts of the decoding procedure in order to try different analyses and to extend the toolbox's functionality. For example, one can easily try different pattern classification algorithms by creating classifier objects that have train and test methods. By running separate analyses using different classifiers one can then assess whether the decoding results are dependent on the particular classifier that is used (Heller et al., 1995; Zhang et al., 1998; Meyers and Kreiman, 2012). The first release of the NDT (version 1.0) comes with two different datasource objects, three feature-preprocessors, three classifiers, one cross-validator, and a number of helper tools (i.e., useful additional functions and objects) that allow one to easily plot results and format the data (see Figure 2). In the future, the toolbox might be expanded to include additional objects.

## Data formats

**Figure 3:**
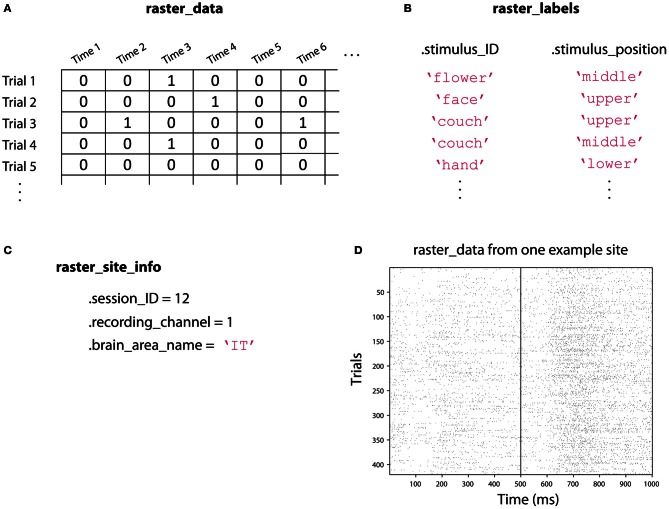
**An illustration showing the three raster-format variables. (A)** raster_data is a [number-of-trials × number-of-time-points] matrix that contains the data from the neural recordings. Here we illustrate spiking data where a one indicates that an action potential occurred at a particular point in time, but this matrix could contain real values such as EEG voltage recordings. **(B)** raster_labels is a structure where each field in the structure contains a [number-of-trials × 1] cell array of strings that indicates which experimental condition occurred on each trial. Here we have two types of labels: .stimulus_ID which indicates which image was shown on a particular trial, and .stimulus_position which indicates where the stimulus was shown on the screen. **(C)** raster_site_info contains any additional useful information about the recorded site. Here we include which recording session the data comes from, which channel was used for the recoding, and the brain area where the recording was made. **(D)** An example of the raster_data from one site from the Zhang–Desimone seven object dataset created using the function imagesc(~raster_data); colormap gray. Spikes are indicated by black marks. Files in raster-format need to be converted into binned-format before they can be used for decoding, which is typically done using the tool create_binned_data_from_raster_data.

In order to use the NDT, we have defined two data formats, called *raster-format*, and *binned-format*. For most experiments, researchers should start by putting data into raster-format (Figure 3). Data that is in raster-format contains three variables: raster_data, raster_labels, and raster_site_info, and data from each site is saved in a separate file that contains these variables (by site we mean data from one functional unit of interest such as one neuron's spiking activity, one LFP channel, one EEG channel, etc.). The variable raster_data is a matrix where each row corresponds to data from one trial, and each column corresponds to data from one time point. The variable raster_labels is a structure where each field contains a cell array that has the labels that indicate which experimental conditions were present on each trial (thus each cell array in raster_labels has as many entries as there are rows in the raster_data matrix)^4^. Finally, the raster_site_info structure contains any additional information about the sites that one wants to store. For example, one could include information about the date that each site was recorded, what brain region a site came from, etc. This information could be used in the decoding procedure to include only sites that meet particular criteria.

To be able to run a decoding analysis using the NDT, one must convert data into binned-format, which is typically done using the function create_binned_data_from_raster_data. Data that is in binned-format is similar to raster-format, in that in contains three variables, which are named binned_data, binned_labels, and binned_site_info. The variables binned_data and binned_labels are cell arrays where each entry in the cell array contains information from one of the raster-format sites, and binned_site_info contains aggregated site information from all the raster_site_info variables. The one difference is that binned_data generally contains information at a lower temporal resolution compared to raster_data (i.e., the matrix in each cell array entry that correspond to data from one site generally has few columns), which allows data from all the sites to be stored in a single cell array. More information about these formats and about the objects that come with the NDT can be found on the website www.readout.info^5^.

## Examples of using the NDT

To illustrate some of the functionality of the NDT, we will use single unit recordings from macaque inferior temporal cortex (IT) that were collected by Ying Zhang in Robert Desimone's lab at MIT. The data come from an experiment in which a monkey viewed a fixation point for 500 ms and then viewed a visual image for 500 ms. On each trial, one of seven different images was shown (car, couch, face, kiwi, flower, guitar, and hand), and each image was presented at one of three possible locations (upper, middle, lower)^6^. These 21 different stimulus conditions were repeated at least 19 times (Zhang et al., 2011). In each recording session, data from 4 to 11 neurons were simultaneously recorded; thus to do an analyses over a larger population of neurons requires the creation of pseudo-populations (i.e., populations of neurons that were recorded separately but treated as if they were recorded simultaneously).

For the purpose of these examples, will assume that the data from each neuron is in raster-format, and that these raster-format files are stored in the directory ZD_7object_raster_data/. We will also assume that information about which object was shown on each trial is in a structure called raster_labels.stimulus_ID, information about the position of where the stimulus was shown is in a structure called raster_labels.stimulus_position, and that there is an additional variable raster_labels.combined_ID_position that contains the combined stimulus and position information (e.g., “car_upper”). The data used in these examples can be downloaded from www.readout.info and there are also more detailed tutorials on the website.

### Decoding basic stimulus information

For our first example analysis, we will decode which of the seven objects was shown, ignoring the position of where the object was presented. To do this we start by converting data from raster-format into binned-format using the function create_binned_data_from_raster_data. The first argument of this function is the name of the directory where the raster-format files are stored, the second argument is a prefix for the saved binned-format file name, the third argument is the bin size over which the data should be averaged, and the fourth argument is the sampling interval over which to calculate these averages. In this example we will create binned data that contains the average firing rates in 150 ms bins that are sampled at 50 ms intervals, and we will save the results in a file called Binned_7object_data_150ms_bins_50ms_sampled.mat. To do this we run the command:


1 binned_file_name = create_binned_data_
    from_raster_data(’ZD_7object_raster_
    data/’, ’Binned_7object_data’, 150, 50);


Next we create a basic_DS datasource, which is a datasource that has the ability to create pseudo-populations. The first argument to basic_DS is the name of the file that has the data in binned-format, the second argument is the name of the specific binned_labels that should be used for the decoding, and the third argument gives the number of cross-validation splits that should be used. When creating this datasource, we will specify that 20 cross-validation data splits should be used which corresponds to the training the classifier on 19 splits and testing the classifier on the remaining split. We will also create a maximum-correlation-coefficient classifier that will be trained and tested on the data generated by the datasource, and a cell array that contains a feature-preprocessor object that will *z*-score normalize the data so that neurons with higher firing rates will not have a larger influence on the classification procedure^7^. These steps can be done using the commands:


2 ds = basic_DS(binned_file_name,
  ’stimulus_ID’, 20);
3 cl = max_correlation_coefficient_CL;
4 fps{1} = zscore_normalize_FP;


The final step in the decoding procedure is to create a cross-validator object, and run the decoding procedure using this object. We will use the standard_resample_CV object, which creates robust results by running the decoding procedure multiple times with different data in the training and test splits, and then averaging the decoding accuracies from these different “resample runs” together^8^ (see Figure 1). This can be done using the commands:


5 cv = standard_resample_CV(ds, cl, fps);
6 DECODING_RESULTS = cv.run_cv_decoding;
7 save(’basic_results’, ’DECODING_RESULTS’)


Once the decoding procedure has been run, we can plot the results using the plot_standard_results_object. We can also add a line at 500 ms indicating the time when the stimulus was shown.


**Figure 4:**
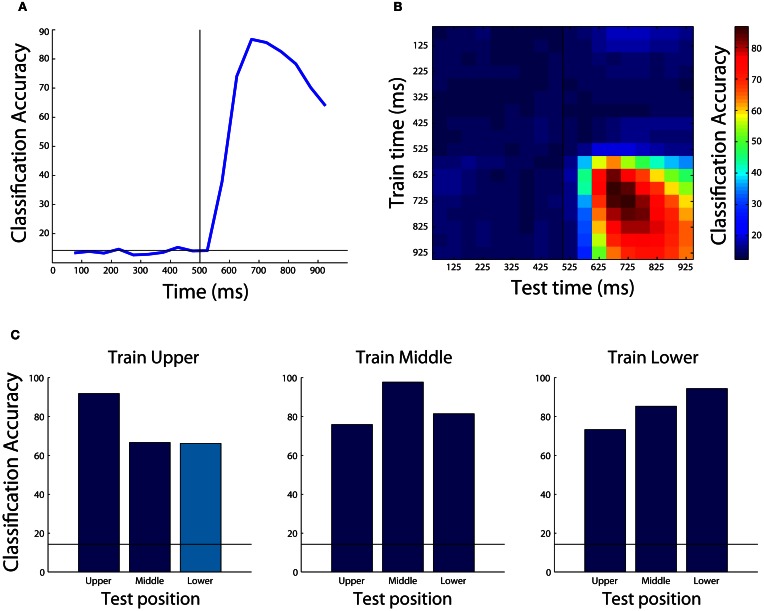
**Examples of results that can be obtained using the Neural Decoding Toolbox. (A)** Basic classification accuracy results from decoding which object was present using the Zhang–Desimone seven object dataset. As can be seen, prior to the onset of the stimulus (black vertical line at 500 ms) the decoding accuracy is at chance (black horizontal line) and it rises quickly after stimulus onset. **(B)** Results examining whether information is contained in a dynamic population code that are obtained by training the classifier at one time period (y-axis) and testing the classifier at a second time period (x-axis). The results here show there are some dynamics to the population code, as can be seen by the fact that the some of the highest decoding accuracies occur along the diagonal of the plot, however, overall there dynamics appear weak compared to those seen in other studies (Meyers et al., 2008, 2012; Crowe et al., 2010; Carlson et al., 2011; Isik et al., 2012). **(C)** Results showing that IT populations are highly invariant to changes in the position of the stimulus. Each subplot shows the results from training the classifier using data from objects shown at one position, and each bar shows the results from test the classifier with data from either the same position or at a different position (the cyan bar shows the results generated by the code in the text). As can be seen, the best results are usually obtained when the classifier is trained and tested with data from the same position, however, even when the classifier is tested with data from a different position, the results are well above chance (black horizontal line) showing that the neural population represents objects similarly across different positions.

8 plot_obj = plot_standard_results_object
  ({’basic_results’});
9 plot_obj.significant_event_times = 500;
10 plot_obj.plot_results;


The results from this analysis are shown in Figure 4A. As can be seen, the decoding accuracy is at chance prior to the onset of the stimulus (since the monkey is not psychic), however, shortly after the stimulus is shown, we are able to decode which object was presented with a peak classification accuracy of around 90%. Different types of results such as normalized rank results, or mutual information can also be plotted by changing the plot_obj.result_type_to_plot field, and there are a number of other properties that can also be modified to change the appearance of the figure.

We can also examine whether information is coded by a dynamic population code (i.e., do different patterns of neural activity code the same information at different latencies in the trial). Previous work showed that information in several brain regions often is contained in a highly dynamic population code (Meyers et al., 2008, 2012; Crowe et al., 2010; Carlson et al., 2011). To test whether information is contained in a dynamic population code, we can plot a temporal cross-training matrix (TCT plot) using the plot_standard_results_TCT_object as follows:


11 plot_obj = plot_standard_results_TCT_
   object(’basic_results’);
12 plot_obj.significant_event_times = 500;
13 plot_obj.plot_results;


The results from this plot are shown in Figure 4B. As can be seen, the information in IT has some slight dynamics (e.g., training at 675 ms and testing at 875 ms leads to lower performance than training and testing at 875 ms), however, overall there is a lot of similarity between the patterns of neural activity across time points in the trial.

### Examining position invariance using the NDT

An important step in solving many tasks faced by intelligent organisms involves creating abstract (or invariant) representations from complex input patterns. For example, in order to act appropriately in social settings, it important to be able to recognize individual people. However, the images of a particular person that are projected on our retinas can be very different due to the fact that the person might be at different distances from us, in different lighting conditions, etc. Thus, at some level in our brain, neural representations must be created that have abstracted away all the details present in particular images to create invariant representations that are useful for action.

A powerful feature of the NDT is that it can be used to test whether a population of neurons has created representations that are invariant to particular transformations. To test whether neural activity is invariant to a transformation, one can train a classifier under one set of conditions, and then test to see if the classifier can generalize to a related set of conditions in which a particular transformation has been applied. The generalization_DS datasouce object is useful for this purpose.

To demonstrate how to use the NDT to do such a “generalization analysis,” we will analyze how invariant representations of objects in the inferior temporal cortex are to translations in the objects' position. In particular, we will examine whether a classifier that is trained using data that was recorded when images were shown in an upper retinal location can discriminate between the same objects when they are shown at the lower location. To do this analysis, we start by creating binned-format data that consists of firing rates averaged over a 400 ms bin starting 100 ms after stimulus onset, and we create the same feature preprocessor and classifier used in the previous example.


1 file_name = create_binned_data_from_raster
  _data(’ZD_7object_raster_data/’, ’Binned_
  7object_data’, 400, 400, 601, 1000);
2 cl = max_correlation_coefficient_CL;
3 fps{1} = zscore_normalize_FP;


To test for position invariance, we use the combined position and stimulus ID labels. Also, to use the generalization_DS we need to create a cell array that lists which label names the classifier should be trained on, and a cell array that lists the label names the classifier should be tested on. Each cell entry in these “training_label_names,” and “test_label_names” cell arrays corresponds to the labels that belong for one class (i.e., training_label_names{1} are the labels that the classifier should be trained on for class 1, and test_label_names{1} are the names that the classifier should predict in order for it to be counted as a correct prediction). We create these cell arrays containing the appropriate labels for training at the upper position and testing at the lower position as follows:


4 id_names = {’car’, ’couch’, ’face’,
  ’kiwi’, ’flower’, ’guitar’, ’hand’};
5 for iID = 1:7
6 training_names{iID} = {[id_names{iID}
  ’_upper’]};
7 test_names{iID} = {[id_names{iID}
  ’_lower’]};
8 end


Now that have we have created cell arrays that list the appropriate training and test labels, we can create the generalization_DS datasouce, which has a constructor that takes the same first three arguments as the basic_DS datasource, and takes the training and test label cell arrays as the last two arguments^9^.


9 ds = generalization_DS(file_name,
  ’combined_ID_position’, 18, training
  _names, test_names);


Finally, we can again use the standard_resample_CV cross-validator to run this decoding analysis.


**Figure A2:**
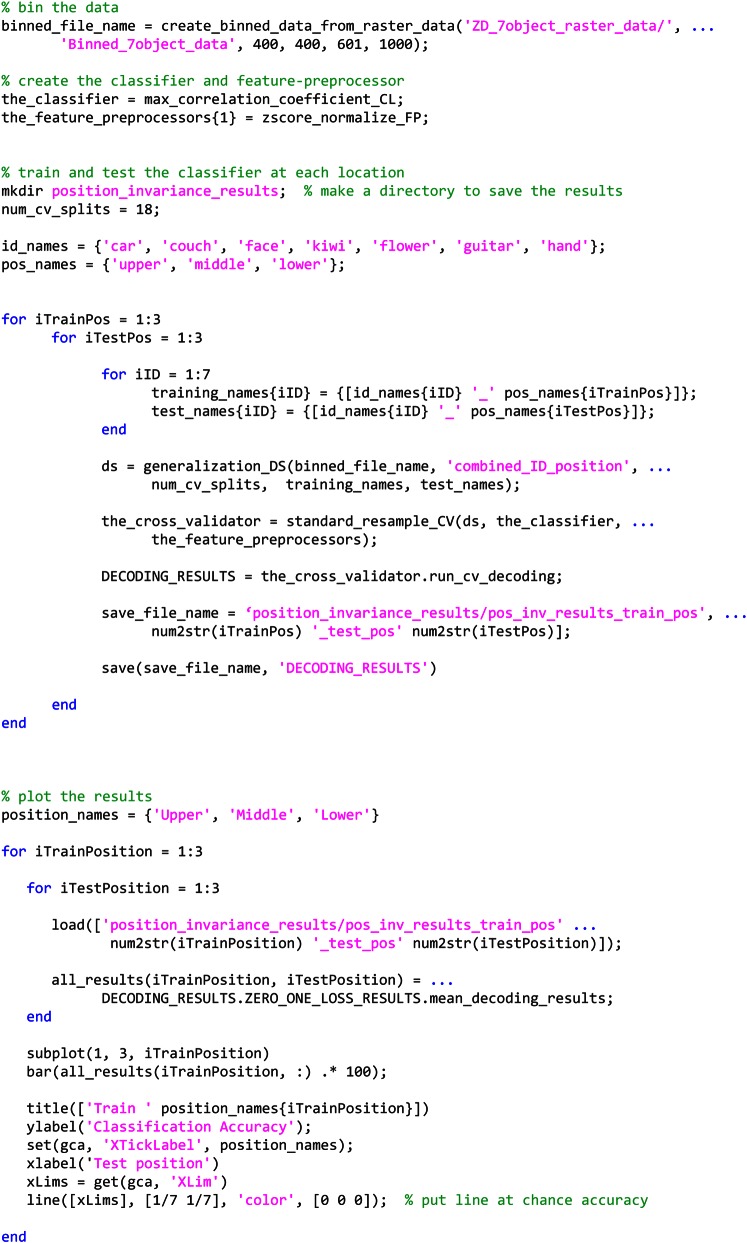
**Full code for training the classifier at one location and testing the classifier at either the same or a different location**.

10 cv = standard_resample_CV(ds, cl, fps);
11 DECODING_RESULTS = cv.run_cv_decoding;


Figure 4C shows the results from training at the upper position and testing at lower position (cyan bar), as well as all the other combinations of results for training at one location and testing either at the same or a different location (blue bars), and Figure A2 shows how to create the full figure. As can be seen, slightly higher decoding accuracies are obtained when the classifier is trained and tested at the exact same location, however, overall very similar performance is also obtained when training and testing at different locations. This indicates that the neural representation in IT is highly invariant to the exact position that a stimulus is shown.

## Comparisons to other decoding toolboxes

At the time of writing this paper, we are aware of two other software packages, the princeton-mvpa-toolbox and PyMVPA, that can also perform decoding analyses. The princeton-mvpa-toolbox is a Matlab toolbox that is designed to analyze fMRI data, and has several useful functions for that purpose such as the ability to import fMRI data, map selective voxels back to their anatomical coordinates, and to perform a search light analysis (Detre et al., 2006). Thus if one is interested in using Matlab to analyze fMRI data, we recommend using this toolbox over the NDT. However, because the princeton-mvpa-toolbox is designed for fMRI data analyses, it is not easy to extend it to analyze other types of neural data that have a temporal component to them (such as neural spiking data, and electroencephalography recordings), thus the NDT is more useful for analyzing such data. The PyMVPA software package is a set of decoding modules written to do decoding analyses in Python. While its most extensive functionality is also geared toward fMRI decoding analyses (it can perform all the functions available in the princeton-mvpa-toolbox), the PyMVPA package also has the ability to handle time series data, and thus it can be used to analyze electrophysiology as well (Hanke et al., 2009). Using Python to analyze data has some advantages over using Matlab including the fact that Python is free and it is a more easily extendable and better organized programming language. However, currently most neuroscience researchers use Matlab as their primary data analysis language, thus we believe the NDT will be valuable to a large number of researchers who are already familiar with Matlab and want to be able to easily run decoding analyses on their data. Additionally, the NDT supports pseudo-populations, allows one to create TCT plots, and allows one to easily do generalization analyses, which are features that are not currently built in to the PyMVPA toolbox. Given that most electrophysiology studies still collect data from only a few neurons at a time, having the ability to create pseudo-populations is critical for being able to analyze most electrophysiology data. Also, the ability to examine neural population coding across time, and the ability to test whether a neural representation is invariant/abstract from specific stimulus conditions are some of the greatest advantages that population decoding methods have over conventional single site analyses. Thus we believe the NDT is a useful addition to the other decoding tools that are currently available.

## Conclusion

In this paper we have described the organization of the NDT. We also have given examples of how the toolbox can be used to decode basic information, and how it can be used to assess more complex questions such as whether information is contained in a dynamic population code and whether information is represented in an abstract/invariant format. While the examples in this paper have focused on analyzing neural spiking activity from experiments that explored questions related to vision, we have also used the toolbox to decode magnetoencephalography signals, local field potentials, and computational model data (Meyers et al., 2010; Isik et al., 2012), so we believe the toolbox should be useful for analyzing a variety of signals and from experiments that analyze questions related to a variety of perceptual (and abstract) modalities.

While we have highlighted some of the key features of the toolbox in this paper, there are several additional properties and functions that we did not describe in detail. For example, if one has a dataset where all the sites were recorded simultaneously, it possible to easily examine whether there is more information in the interaction between neurons by comparing the results when the ds.create_simultaneously_recorded_population property in the basic_DS or generalization_DS is set to different values (Franco et al., 2004; Latham and Nirenberg, 2005; Anderson et al., 2007). We refer the reader to the website readout.info in order to learn more about all the features available in toolbox. Additionally, because the toolbox is designed in a modular manner it is easy to expand its functionality, and we aim to continue to add new features in the future. We also hope that the data formats we defined will be useful for sharing data and will enable the development of new data analysis tools that can all be easily applied to the same data.

The code for the NDT is open source and free to use (released under the GPL 3 license). We do, however, ask that if the toolbox is used in a publication, that the data that has been used in the publication is made available within 5 years after the publication since such sharing of data helps in the development of new data analysis tools and that could potentially lead to new discoveries (Teeters et al., 2008). Population decoding analyses have several advantages over other data analysis methods (particularly in terms of the ability to assess abstract information and dynamic coding). It is our hope that by releasing this toolbox, population decoding methods will be more widely used and that this will lead to deeper insights into the neural processing that underlies complex behaviors.

### Conflict of interest statement

The author declares that the research was conducted in the absence of any commercial or financial relationships that could be construed as a potential conflict of interest.

## Footnotes

^1^The number of times that each condition needs to be presented (and the number of sites that need to be recorded) depend on the type of data being analyzed. For neural spiking activity, we have found we can get meaningful results with a little as three repeated trials of each condition, and with recordings from as few as 40 neurons, although to get more reliable results we recommend at least eight repetitions of each experimental condition, and at least 100 recorded neurons.

^2^The toolbox does not require that the data from the multiple sites was recorded simultaneously, which makes it particularly well suited for analyzing electrophysiology data given that most electrophysiology experiments currently only record from a small number of neurons at a time.

^3^If there is no useful preprocessing information that should be saved, then this method should just return an empty matrix.

^4^The variable raster_labels is defined as a structure to allow one to decode multiple types of information from the same data; see ’Examples of using the NDT’ below.

^5^Note that the extension of the domain of the website is “.info” (so a “.com”, “.org” or any other extension should not be included when entering this URL in a web browser).

^6^The experiment also contained 3 object trials and the monkey's attention was manipulated, however for the purpose of illustrating the usefulness of the toolbox, we are only using data from single object trials.

^7^A preprocessing pipeline can be built by creating multiple feature preprocessors and by placing them in a cell array in the desired order.

^8^The parameter cv.num_resample_runs determines how resampling runs to use (the default value is 50).

^9^The training and test label names are cell arrays in order to allow one to set multiple different labels as belonging to the same class. For example, if we wanted to train the classifier using all data from the upper and middle locations we could define use training_names{1} = {’car_upper’, ’car_middle’}; training_names{2} = {’couch_upper’, ’couch_middle’};,..., training_names{7} = {’hand_upper’, ’hand_middle’}.
